# Operative Treatment of Hepatic Hydatid Cysts: A Single Center Experience in Israel, a Nonendemic Country

**DOI:** 10.1155/2013/276807

**Published:** 2013-09-23

**Authors:** Daniel Maoz, Franklin Greif, Jacob Chen

**Affiliations:** ^1^Department of Surgery A, Rabin Medical Center, Beilinson Campus, Petach Tikva 49100, Israel; ^2^Sackler Faculty of Medicine, Tel Aviv University, P.O. Box 39040, Tel Aviv 6997801, Israel

## Abstract

*Background*. Hydatid cyst disease is a zoonosis caused by *Echinococcus* genera. The disease is endemic to certain rural areas in the world. Operative treatment is the main component in curing hydatid cysts of the liver. *Objective*. Describing the unique characteristics of the hydatid cyst patients in Israel, a nonendemic country. *Methods*. Data was collected form 29 patients treated operatively in Rabin Medical Center from 1994 to 2007. *Results*. The study included 18 females and 11 males with an average age of 54.9 years. Fifty-two% of the patients immigrated as children from Arab countries to Israel, 21% were Arab-Israelis leaving in the north and center of Israel, and 24% immigrated from the former Communist Bloc. Pericystectomy was performed in 20/29, and cyst unroofing was performed in 9/29. Hydatid cysts average size was 10.7 cm, and the cysts were located in the right or left or involved both lobes in 62%, 28%, and 10% of the lesions, respectively. Postoperative mortality occurred in one case, and severe morbidity occurred in 4 patients. *Conclusions*. Hydatid cyst disease in Israel is uncommon and is mostly seen in distinct 3 demographic groups. Despite the relatively low patient volume, good results in terms of morbidity, mortality, and recurrence were achieved.

## 1. Introduction

Hydatid cyst disease is a zoonosis caused mainly by the cestode genus *Echinococcus granulosus* (and to much lesser extent, other 3 *Echinococcus* genera). The incidence of this disease is strongly correlated with certain geographic areas in the world. It is endemic to many rural areas where humans are in close contact with the different hosts in the complex life cycle of the parasite. Livestock (e.g., sheep and cattle) are the intermediate host, and small carnivores (e.g., dogs) are the definitive hosts. In humans, like in herbivores, the ingested eggs hatch, and then a hydatid cyst filled with larvae is formed. The larvae cannot develop into its adult form unless it is digested by a carnivore.

In 50%–70% of hydatid disease cases, the liver is involved [1]. Hydatid cysts are asymptomatic for long periods of time in many cases [2] and are diagnosed as an incidental finding or when they become symptomatic. The cysts tend to grow and cause local mass effect, or in other cases they can get infected. Cyst growth can also cause rupture and peritoneal dissemination or anaphylaxis when the cyst content is exposed to the immune system. A connective tissue capsule, a pericyst, is formed around the parasite to seclude it from the host. The pericyst plays an important role as a plane for surgical excision of the germinal layer.

The management of hydatid cyst of the liver usually relies on a surgical procedure as the main component of therapy [3, 4]. The operative approach is based on evacuation of the cyst content as well as the germinal layer lining the cyst while great care is devoted for avoidance of spillage of the cyst's content. Surgery can be accompanied with albendazole or mebendazole therapy [5, 6]. Percutaneous drainage has been proposed as a safe and effective alternative for a surgical procedure in patients that cannot or refuse to have surgery [7, 8]. Medical treatment alone is insufficient [4].

The aim of this study is to define the unique features of patients managed surgically for liver hydatid disease in Israel. Furthermore, by reviewing our surgical experience we intend to try and compare a pericystectomy with cyst unroofing.

## 2. Patients and Methods

The computerized and paper archives of patients treated surgically for hepatic hydatid cyst were reviewed. All cases in Rabin Medical Center, a large tertiary referral center in Israel from 1994 to 2007, were included. Data were collected on patient's demographic characteristics, signs and symptoms on presentation, clinical and laboratory findings, management, outcome, and pathology findings. Hospital stay, operative blood transfused, severe morbidity, and cyst size for the two different surgical techniques were compared using a Student's *t*-test.

## 3. Results

Twenty-nine patients, 18 females and 11 males, underwent an operation for hepatic hydatid cyst in Rabin Medical Center from 1994 to 2007 (see patients' characteristics in Table 1). The average age at operation was 54.9 ranging from 17 to 85 years.

Fifty-two percent of the study population immigrated to Israel from Arab countries (8 from Iraq, 3 from Iran, 2 from Morocco, and 1 from Algiers and Libya each). These immigrants arrived to Israel in the 1950s and 1960s when their average age was 12.4 years old (ranging from 1–20 years). Another group of patients was immigrants from Eastern European countries and the former Soviet Union (2 from Romania, 2 from Russia, and 1 from Uzbekistan and Azerbaijan each) composing 21% of the study population. The third group of patients comprised 24% of the patients and was Israeli-Arabs from the north and center of Israel. Three of these patients were from a single town. All patients underwent abdominal ultrasound and/or CT scan prior to surgery.

Sixty-nine percent (20/29) of the operations were pericystectomy where the entire hydatid cyst is excised and thirty-one percent (9/29) of the operations were unroofing of the cyst in which the cyst is decompressed (see surgical's characteristics in Table 2); it is washed with scolicidal agent fluid, and the germinal layer is removed. No statistical significant difference was found between the two surgeries regarding the length of hospital stay, operative blood transfused, severe morbidity, or cyst size.

In five cases the operation was urgent due to infected cyst in three patients and invasion to the chest cavity and pulmonary symptoms in 2 other patients. In 85% of the patients, a single cyst was found. In 62% the hydatid cysts were only in the right lobe of the liver, 28% involved only the left lobe, and in 10% the lesion was bilateral.

The average size of the cysts was 10.7 cm ranging from 1 cm to 20 cm. Intra-abdominal operative spillage of the cyst content occurred in 7 cases. In four patients, the operations were a second operation subsequent to recurrence of hydatid disease that was operated elsewhere previously.

One case (3.4%) of operative mortality (30 days mortality) was recorded. The patient underwent urgent operation due to sepsis subsequent to biliary invasion. The hydatid cyst was attached to the vena cava. During surgery, the patient suffered severe hemorrhage and died 7 days later. The rate of minor postoperative morbidity was 37.9%, and severe morbidity occurred in 13.8%. The later was composed of 2 cases of biliary leaks, one case of pneumothorax due to thoracic invasion, and one case of anaphylaxis due to cyst rupture.

Antihelminthic medical therapy (albendazole/mebendazole) was administered to all elective patients 3 months prior to surgery. No known recurrences of hydatid cyst were noted in our study population.

## 4. Discussion

This study included 29 patients that underwent surgery for liver hydatid cyst in Rabin Medical Center, Israel, during a 14-year period. To the best of our knowledge, this is the first report of hydatid cyst surgical series in Israel and only one of a small number of reports [9] describing hydatid cysts in nonendemic countries. Thus, the relatively small population in our study is a representation of the low occurrence of hydatid disease in Israel. This notion is further supported since Rabin Medical Center is a large tertiary referral center for hepatobiliary surgery with a large catchment area. Many Mediterranean and Middle Eastern countries (e.g., Turkey, Spain, Iran, etc.) are endemic to hydatid disease [10–13]; however, it does not seem to be the case in Israel which resembles Western and Northern European countries in terms of the disease's incidence. It can be safely assumed that this is due to regulation and industrialization of cattle farming in Israel, good supervision by the veterinarian services, and paucity of freely roaming small carnivores.

The demographic composition of the study population is interesting. It can be divided to 3 distinctive groups as follows.

1. Jewish immigrants from Arab countries that arrived to Israel as children or young adults (average age 12.4 years). They had only few years of exposure at their country of birth with a period of few decades until appearance of symptoms and reaching a diagnosis of their hydatid disease. This group best demonstrates the lengthy and relatively nonvirulent natural course of uncomplicated hydatid disease as was previously described [2].
2. Jewish immigrants from the former Communist countries. These patients arrived from communist countries mostly in the late 1980s and early 1990s. This group was characterized by different age groups and exposure periods prior to immigration to Israel.
3. Israeli-Arabs from the central and northern parts of Israel. Usually known for its rural nature, in most cases this group has closer connections to nonindustrialized livestock farming. The patients in this group were relatively young at presentation. This may be due to more intense exposure.

All of the patients in the study could be ascribed to one of the 3 demographic groups. By so, we describe here an important risk factor unique to hydatid cyst patients in Israel. This is a derivative of the unique sociodemographic structure in Israel.

In this study, complete resection of the hydatid cyst was attempted by performing pericystectomy when it was technically possible. The favorable ratio between pericystectomy and unroofing performed in our study demonstrates this approach. In some cases of very large cysts and cysts adjacent to vascular or biliary structures or severe abdominal adhesions, pericystectomy could not be performed, and unroofing of the cyst was chosen. In cyst unroofing stringent precautions were taken to avoid intra-abdominal spillage of the cyst content and attention was directed to meticulous scraping of the entire germinal layer.

Many authors in the recent literature agree that radical resection of the hydatid cyst should be preferred when feasible [14–17], although some believe complete resection does not correlate with superior results [18, 19]. In accordance with the mentioned reports we did not find a statistically significant difference in cyst size necessitating the two operations nor have we found a difference in severe complication rate, blood transfusions required in the operation room, or length of hospital stay. This could potentially be ascribed to small sample sizes or distinct selection bias since surgical technique was chosen based on technical difficulties per definition.

The mild complication rate was 37.9% and consisted mainly of postoperative mild anemia, uncomplicated fever, and atelectasis. The severe complication rate was 13.8%. We had one case of post surgical mortality. These complication rates are comparable or slightly higher than results of medical centers in endemic countries with higher volume of hydatid cysts [15, 20, 21]. The slightly higher mild postoperative morbidity rate might be caused by different inclusion criteria of mild morbidity not including mild routine postoperative morbidity such as mild anemia and transient uncomplicated fever. The severe morbidity rate demonstrates that even in nonendemic areas active hepatobiliary surgery service funneling hydatid cyst cases can maintain good surgical results.

The relatively small population size in our study is a double-edged sword. On one hand, it limits the ability to reach statistically based conclusions, but, on the other, it holds the key in describing the features of hydatid disease in a nonendemic area. Another possible limitation of this study is a lack of adequate long-term followup due to relatively short surgical followup. To the best of our knowledge no hydatid recurrences occurred. It can be safely assumed that the report rate on recurrence is close to 100%.

In conclusion, hydatid cyst disease in Israel is infrequent. Patients most likely belong to one of 3 demographic groups: immigrants from Arab countries, the former Communist countries, or Israeli-Arabs. The preferred surgical technique is cyst pericystectomy when possible. Surgical treatment is undoubtedly reasonable in terms of morbidity, recurrence, and mortality.

## Figures and Tables

**Table 1:** Study population characteristics.

Gender	
Male	11
Female	18
Age (yrs)	54.9 ± 16.8
Demographic groups	
Immigration from the former Communist Bloc	6
Immigration from Arab countries	15
Israeli-Arabs (born in Israel)	7
Israeli-Jews (born in Israel)	1
Extrahepatic involvement	3

Data presented as mean ± standard deviation.

**Table 2:** Study population surgical characteristics.

Surgical procedure	
Pericystectomy	20
Cyst Unroofing	9
Type of surgery	
Elective	24
Urgent	5
Number of cysts	1.2 ± 0.5
Cyst size (cm)	10.7 ± 4.6
Cyst location	
Right	18
Left	8
Bilateral	3
Patients requiring blood products	3
30 days of mortality	1
Mild morbidity	11
Severe morbidity	4

Data presented as mean ± standard deviation.
